# Urinary sodium/potassium ratio as a screening tool for hyperaldosteronism in men with hypertension

**DOI:** 10.1038/s41440-021-00663-9

**Published:** 2021-05-17

**Authors:** Hiroyoshi Segawa, Akane Higashi, Izuru Masuda, Kengo Yoshii, Toshiyuki Iwahori, Hirotsugu Ueshima

**Affiliations:** 1 Graduate School of Life and Environmental Sciences, Kyoto Prefectural University, Kyoto, Japan; 2 Center for Epidemiologic Research in Asia, Shiga University of Medical Science, Shiga, Japan; 3 Takeda Hospital Medical Examination Center, Kyoto, Japan; 4 Department of Mathematics and Statistics in Medical Sciences, Kyoto Prefectural University of Medicine, Kyoto, Japan; 5 Department of Public Health, Shiga University of Medical Science, Shiga, Japan

**Keywords:** Hyperaldosteronism, Hypertension, Urinary sodium/potassium ratio

## Abstract

Among individuals with hypertension, the prevalence of secondary hypertension has been reported to be ≈10%. More than half of individuals with secondary hypertension have associated hyperaldosteronism. However, given the current clinical environment, these patients often remain undiagnosed. We hypothesized that the urinary sodium/potassium ratio (Na/K) could be used as a simple, low-cost method of screening for hyperaldosteronism among individuals with hypertension in primary care and health examination settings. We recruited hypertensive individuals aged 30–69 years old who were not taking any antihypertensive medications from among participants in health examinations. Urinary Na and K were measured using second morning urine samples, and the plasma aldosterone concentration (PAC) was also measured. We evaluated the association of the second morning urine Na/K ratio (SMU Na/K) with a high PAC, defined as ≥90th percentile (24.3 ng/dL), using receiver operating characteristic (ROC) curves. Overall, 160 participants (108 men and 52 women) with a mean age of 54.3 years were eligible for this study. The area under the ROC curve for the relationship between SMU Na/K and high PAC was 0.77 (95% confidence interval [CI]: 0.59–0.95) in men and 0.64 (95% CI: 0.36–0.93) in women. In men, SMU Na/K values <1.0 could detect hyperaldosteronism with a sensitivity of 45.5%, a specificity of 97.9%, a positive predictive value of 71.4%, and a negative predictive value of 94.1%. The use of the urinary Na/K ratio may be appropriate as a method of screening for hyperaldosteronism in hypertensive men.

## Introduction

Aldosterone is a mineralocorticoid hormone that regulates blood pressure and sodium (Na)–potassium (K) exchange in the distal tubules and collecting ducts of the kidney. Aldosterone affects blood pressure increases, Na reabsorption, and K excretion. In some diseases, such as primary aldosteronism and renovascular hypertension, aldosterone is inappropriately secreted, which leads to hypertension [1, 2]. These conditions are called hyperaldosteronism or aldosterone excess.

The prevalence of primary aldosteronism, a representative cause of hyperaldosteronism, has been reported to be 4.6–16.6% among individuals with hypertension worldwide [3]. In Japan, the prevalence of secondary hypertension has been reported to be 9.1%; more than half of patients with secondary hypertension have associated hyperaldosteronism [2]. In a meta-analysis, it was reported that patients with primary aldosteronism have a high risk of cardiovascular diseases (CVDs) [4]. Furthermore, hyperaldosteronism is currently considered to be a risk factor for subclinical atherosclerosis, CVD mortality, and all-cause mortality [5–7] regardless of the presence of secondary hypertension. Although hyperaldosteronism is a common cause of antihypertensive treatment resistance [8], it is treatable with specific therapies, such as surgical treatment for aldosterone-producing adenoma and endovascular treatment for renovascular hypertension caused by fibromuscular dysplasia [9]. Mineralocorticoid receptor antagonists can be used as a noninvasive treatment for people with hypertension who have hyperaldosteronism [8]. Thus, early diagnosis and treatment are important for hyperaldosteronism.

Measuring plasma renin activity (PRA) and the plasma aldosterone concentration (PAC) is essential for the detection and classification of hyperaldosteronism. For example, primary aldosteronism is suspected when the PAC/PRA ratio is >20 and the PAC is >12–15 ng/dL, according to the Japanese guidelines for the management of hypertension [9, 10]. Various methods of identifying primary aldosteronism have been proposed [11, 12]. The Japan Endocrine Society recommends the measurement of PAC and PRA in all patients initially diagnosed with hypertension [10]. However, these measurements are cumbersome and expensive and often omitted in primary care settings, which results in the underdiagnosis of hyperaldosteronism [5]. Thus, easy, practical, and inexpensive screening methods that can be used in primary care or health examination settings are needed.

Because hyperaldosteronism causes excessive Na reabsorption and K excretion, we hypothesized that individuals with a high PAC have a low urinary Na/K ratio. Urinary Na/K has been used as an indicator of the PAC in the clinical environment [13]. However, the usefulness of the spot urinary Na/K ratio for detecting a high PAC has not been evaluated. The aim of this study was to assess whether the urinary Na/K ratio can be used to identify individuals with a high PAC from those with hypertension.

## Methods

### Participants and measurements

**Fig. 1:**
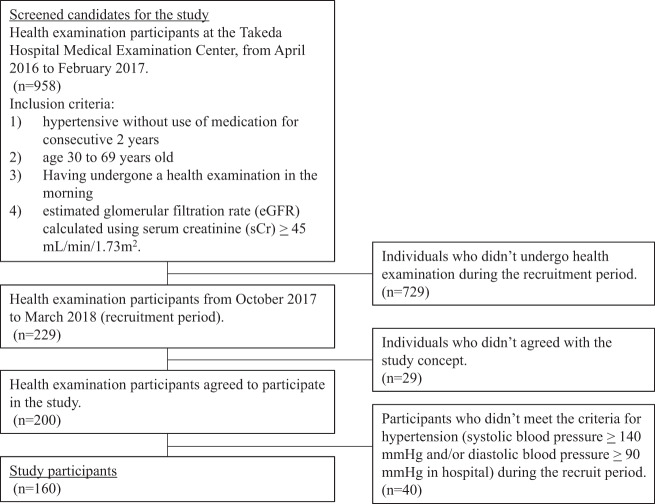
Flow diagram of study participant recruitment. We recruited hypertensive individuals aged 30 to 69 years old who were not taking any antihypertensive medications from among participants in health examinations. Overall, 160 participants (108 men and 52 women) were eligible for inclusion in this study

First, we ascertained the number of candidates for inclusion in the study from among the participants in health examinations at the Takeda Hospital Medical Examination Center from April 2016 to February 2017. Individuals were considered candidates if they met the following criteria: (1) hypertension without the use of medication for two consecutive years, (2) aged 30–69 years old, (3) underwent a health examination in the morning, and (4) available estimated glomerular filtration rate (eGFR) calculated using serum creatinine (sCr) ≥45 mL/min/1.73 m^2^. Second, we sent recruitment documents to candidates who underwent a health examination from October 2017 to March 2018 (recruitment period). As a result, 200 individuals (135 men and 65 women) agreed to participate in our study (Fig. 1). It was confirmed that none of the participants were taking antihypertensive medications. Participants who did not meet the criteria for hypertension (systolic blood pressure ≥140 mmHg and/or diastolic blood pressure ≥90 mmHg in the hospital) during the recruitment period were excluded from the present study.

The gold standard method for measuring excreted Na or K is a 24-h urine collection test. However, this method is cumbersome; therefore, 24-h urinary Na excretion is often estimated using the concentrations of Na and creatinine in second morning urine [14]. Similarly, a previous report showed that the urinary Na/K ratio in second morning urine is closer to that in 24-h urine than that in first morning urine [15]. Therefore, we used the second morning urine Na/K ratio (SMU Na/K) in this study. Participants were instructed to void their first morning urine soon after awakening, and second morning urine was collected on-site during their health examination. The Na/K ratio in each urine sample was calculated using the Na and K concentrations. Laboratory tests associated with the health examination were conducted at the laboratories of Kouseikai Takeda Hospital and Hoken Kagaku Nishi-Nihon. PRA and the PAC were measured using a radioimmunoassay. Na and K were assessed using an electrode method, and sCr was assessed using an enzymatic method. The assay kit used for the measurement of the PAC was the SPAC-S Aldosterone Kit (TFB, Inc. Tokyo, Japan). We calculated the eGFR using the following equation: eGFR (mL/min/1.73 m^2^) = 194 × sCr (mg/dL)^−1.094^ × age^−0.287^ × 0.739 (for women) [16]. Blood sampling was performed after 10 min of rest in a seated position. Baseline blood pressure was measured on-site twice by clinical staff using an automated sphygmomanometer after 10 min of rest in a seated position. The mean of the two blood pressure measurements was calculated and used for each participant. Body mass index was calculated as the weight divided by the height squared (kg/m^2^). In the present study, diabetes mellitus was defined as a glycated hemoglobin level of 6.5% or higher, the use of diabetes medication, or both.

We asked all participants to complete a self-administered questionnaire to collect information regarding hypertension onset, medication, and lifestyle. The intake frequencies for fruit, vegetables, legumes, and salty food were reported by participants, according to the following response options: 3/day, 2/day, 1/day, 3–5/week, 1–2/week, or rarely; the frequency of sweating was also reported according to the following response options: >5/week, 3–4/week, 1–2/week, or rarely. Drinking habits were reported according to the following response options: every day, sometimes, or rarely. Habitual skipping of breakfast (≥3/week), current smoking, and sweating the day before the examination were reported as yes or no.

### Statistical analysis

First, we described participants’ physical characteristics and lifestyles and compared these between men and women. We also described participants’ physical characteristics and lifestyles stratified by PAC level (≥ or <90th percentile value) and compared them separately for each sex. Student’s *t*-test was used for continuous variables, and either the chi-square test or Fisher’s exact test was used for categorical variables. Second, we assessed the associations of the SMU Na/K and other biologically reasonable variables with the PAC using simple and multiple linear regression analysis. We also evaluated the interactive effect of the SMU Na/K and sex on the PAC. In this analysis, the SMU Na/K was log transformed because of its skewed distribution. Multiple regression analysis model 1 was adjusted for all variables used in the simple linear regression analysis, and multiple regression analysis model 2 was adjusted for variables with *p* value < 0.10 in multiple regression analysis model 1. Third, we used receiver operating characteristic (ROC) curves to explore the relationship between the SMU Na/K and a high PAC, defined as ≥90th percentile. Age and serum K were also added to the logistic regression model used to generate the ROC curves to assess the impact of those covariates. We identified a reasonable cutoff point for the SMU Na/K for the detection of individuals with a high PAC. We then evaluated the sensitivity and specificity of the SMU Na/K using this cutoff point. As a sensitivity analysis, we performed a ROC curve analysis from which we excluded 16 participants who had low levels of sodium excretion (≤10th percentile value) using the Kawasaki equation [14]. Analyses were performed separately for each sex because there was a significant interactive effect of the log SMU Na/K and sex on the PAC. All *p* values were two sided, and *p* <0.05 was regarded as statistically significant. Statistical analyses were performed using JMP version 14.3.0 (SAS Institute Inc., Cary, NC, USA) and R version 3.4.3 (R Foundation for Statistical Computing, Vienna, Austria).

## Results

### Descriptive statistics of study participants

Overall, 160 participants (108 men and 52 women) were eligible for inclusion in this study (Fig. 1). Participants’ physical and lifestyle characteristics are shown in Table 1. The mean ages for men and women were 53.2 and 56.7 years, respectively. The mean SMU Na/K values were 2.75 and 2.82 mmol/mmol, and the mean PACs were 16.8 and 17.3 ng/dL, respectively. No participants had an eGFR <45 mL/min/1.73 m^2^. When stratified by sex, women tended to have a healthier lifestyle, with greater frequencies of the intake of fruit, vegetables, and legumes, and a lower frequency of skipping breakfast. The proportions of participants who smoked and drank were higher in men than in women.

**Table 1:** Physical and lifestyle characteristics of participants, 2017–2018

	Total (*n* = 160)	Men (*n* = 108)	Women (*n* = 52)	*p* value
Age (years)	54.3 (8.0)	53.2 (8.1)	56.7 (7.4)	<0.01
Height (m)	1.67 (0.09)	1.72 (0.05)	1.57 (0.06)	<0.01
Body weight (kg)	70.6 (13.9)	75.0 (12.6)	61.5 (11.9)	<0.01
Body mass index (kg/m^2^)	25.2 (3.9)	25.3 (3.8)	24.9 (4.2)	0.53
Systolic blood pressure (mmHg)	148 (12)	146 (12)	151 (11)	<0.01
Diastolic blood pressure (mmHg)	96 (8)	98 (6)	93 (9)	<0.01
Urinary sodium (mmol/L)^a^	147.6 (50.4)	150.2 (52.7)	142.2 (45.3)	0.32
Urinary potassium (mmol/L)^a^	63.3 (26.8)	65.5 (28.0)	58.9 (23.7)	0.12
Urinary sodium/potassium ratio (mmol/mmol)^a^	2.77 (1.55)	2.75 (1.60)	2.82 (1.46)	0.78
Plasma aldosterone (ng/dL)	17.0 (5.4)	16.8 (5.5)	17.3 (5.3)	0.59
Plasma renin activity (ng/mL/hr)	1.10 (0.88)	1.25 (0.94)	0.78 (0.63)	<0.01
Serum potassium (mmol/L)	4.2 (0.3)	4.3 (0.3)	4.2 (0.3)	0.11
Creatinine (μmol/L)	70.2 (14.3)	76.9 (11.9)	56.4 (7.0)	<0.01
eGFR (mL/min/1.73m^2^)	74.8 (12.7)	74.5 (13.2)	75.3 (11.9)	0.71
Sweating the day before the examination	30 (18.9)	24 (22.2)	6 (11.8)	0.18
Sweating ≥5/week	18 (11.3)	13 (12.0)	5 (9.6)	0.85
Vegetables intake ≥1/day	132 (82.5)	82 (75.9)	50 (96.2)	<0.01
Fruit intake ≥1/day	46 (28.8)	27 (25.0)	19 (36.5)	0.19
Legumes intake ≥1/day	60 (37.5)	34 (31.5)	26 (50.0)	0.04
Salty food intake ≥1/day	37 (23.1)	26 (24.1)	11 (21.2)	0.83
Skipping breakfast ≥3/week	31 (19.4)	26 (24.1)	5 (9.6)	0.05
Diabetes mellitus	12 (7.5)	11 (10.2)	1 (1.9)	0.11
Current smoker	24 (15.0)	23 (21.3)	1 (1.9)	<0.01
Drinking				<0.01
-Every day	73 (45.6)	60 (55.6)	13 (25.0)	
-Sometimes	42 (26.3)	34 (31.5)	8 (15.4)	
-Rarely	45 (28.1)	14 (13.0)	31 (59.6)	

Continuous variables are described as mean (standard deviation); categorical variables are described as *n* (%)

*eGFR* estimated glomerular filtration rate

^a^Measured using second morning urine

Note: *p* values are calculated using Fisher’s exact test for dichotomic variables, χ^2^ test for multiple categorical variable (drinking status) and *t*-test for continuous variables

In the analysis stratified by PAC level, the urinary Na and SMU Na/K were significantly lower and the PRA was significantly higher in the high-PAC group among men. The serum K level was significantly lower in the high-PAC group among women (Supplementary Table 1).

### Linear regression analyses

Supplementary Table 2 shows the associations of the variables with the PAC in the entire study population. There was a significant interactive effect of the log SMU Na/K and sex on the PAC. Table 2 shows the associations of the variables with the PAC stratified by sex. In the simple regression analysis, age, sweating ≥5/week, and the log SMU Na/K were significantly associated with the PAC in men; the serum K level also tended to show an association. In multiple regression analysis, the serum K level, vegetable intake frequency ≥1/day and the log SMU Na/K were significantly associated with the PAC in men in models 1 and 2. Neither the log SMU Na/K nor other variables were associated with the PAC in women.

**Table 2:** Associations of variables with the plasma aldosterone concentration stratified by sex

		Simple regression analysis			Model 1			Model 2		
		Regression coefficient	(95% CI)	*p* value	Regression coefficient	(95% CI)	*p* value	Regression coefficient	(95% CI)	*p* value
Men (*n* = 108)	Age (yr)	−1.41	(−2.68, −0.14)	0.03	−1.17	(−2.45, 0.12)	0.08	−1.24	(−2.39, −0.08)	0.04
	Body mass index (kg/m^2^)	2.05	(−0.73, 4.82)	0.15	0.94	(−1.75, 3.64)	0.49	–		
	Systolic blood pressure (mmHg)	−0.30	(−1.21, 0.61)	0.51	0.08	(−0.83, 0.98)	0.87	–		
	eGFR (mL/min/1.73m^2^)	−0.43	(−1.23, 0.37)	0.29	−0.57	(−1.39, 0.25)	0.17	–		
	Serum potassium (mmol/L)	−34.33	(−69.38, 0.73)	0.05	−42.58	(−79.39, −5.77)	0.02	−39.43	(−71.32, −7.54)	0.02
	Sweating ≥ 5/week	34.76	(3.15, 66.36)	0.03	15.12	(−15.45, 45.70)	0.33	–		
	Vegetables intake ≥ 1/day	14.42	(−10.01, 38.85)	0.24	23.62	(0.36, 46.88)	0.05	23.34	(1.65, 45.03)	0.04
	Fruit intake ≥ 1/day	−1.36	(−25.63, 22.92)	0.91	−5.54	(−27.68, 16.59)	0.62	–		
	Salty food intake ≥ 1/day	0.46	(−24.13, 25.05)	0.97	−1.70	(−24.41, 21.01)	0.88	–		
	Drinking									
	Everyday vs rarely	−17.99	(−52.39, 16.42)	0.30	−8.95	(−42.04, 24.13)	0.59	–		
	Sometimes vs rarely	−26.53	(−58.69, 5.63)	0.10	−11.23	(−43.40, 20.94)	0.49	–		
	log SMU Na/K	−38.25	(−54.99, −21.50)	<0.01	−36.94	(−54.26, −19.62)	<0.01	−40.24	(−56.20, −24.28)	<0.01
Women (*n* = 52)	Age (yr)	−0.38	(−2.40, 1.64)	0.71	−0.20	(−3.36, 2.95)	0.90	−0.46	(−2.60, 1.68)	0.67
	Body mass index (kg/m^2^)	0.70	(−2.87, 4.27)	0.70	0.98	(−3.53, 5.50)	0.66	–		
	Systolic blood pressure (mmHg)	0.22	(−1.09, 1.52)	0.74	0.51	(−1.30, 2.32)	0.57	–		
	eGFR (mL/min/1.73m^2^)	0.22	(−1.04, 1.48)	0.73	0.42	(−1.56, 2.40)	0.67	–		
	Serum pottasium (mmol/L)	−19.82	(−65.25, 25.60)	0.38	−18.70	(−73.29, 35.88)	0.49	−24.66	(−73.19, 23.87)	0.31
	Sweating ≥5/week	−2.38	(−52.81, 48.06)	0.92	−23.11	(−90.49, 44.27)	0.49	–		
	Vegetables intake ≥1/day	−18.42	(−95.56, 58.72)	0.63	−46.51	(−148.50, 55.48)	0.36	−24.46	(−108.21, 59.30)	0.56
	Fruit intake ≥1/day	4.61	(−26.24, 35.46)	0.77	8.98	(−38.56, 56.53)	0.70	–		
	Salty food intake ≥1/day	-4.72	(−41.11, 31.66)	0.80	−9.18	(−54.99, 36.63)	0.69	–		
	Drinking									
	Everyday vs rarely	11.52	(−30.94, 53.98)	0.59	21.06	(−36.83, 78.95)	0.47	–		
	Sometimes vs rarely	−13.77	(−49.15, 21.61)	0.44	−11.99	(−56.94, 32.96)	0.59	–		
	Log SMU Na/K	2.46	(−28.54, 33.45)	0.87	−15.25	(−60.27, 29.78)	0.50	−2.38	(−35.50, 30.75)	0.89

*eGFR* estimated glomerular filtration rate, *SMU Na/K* second morning urine sodium/potassium ratio, *CI* confidence interval

Multiple regression analysis model 1 is adjusted for all the above variables

Multiple regression analysis model 2 is adjusted for age, serum potassium, vegetable intake ≥1/day, and log SUM Na/K. The *R*^*2*^ was 0.241 in men

### ROC curve analyses

**Fig. 2:**
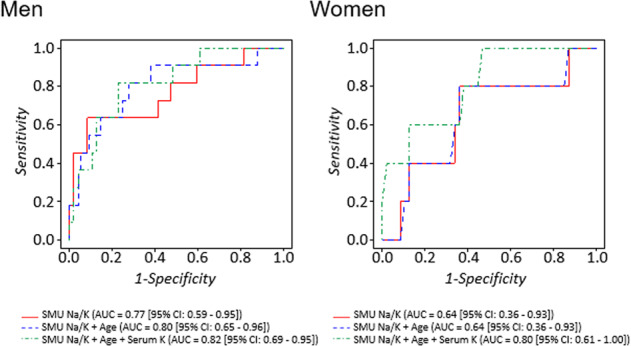
ROC curve analysis of the association of the SMU Na/K with a high PAC (≥90th percentile) stratified by sex (108 men and 52 women).The SMU Na/K was significantly associated with a high PAC in all models for men but only in the model including age and the serum K level (dashed line) in women. ROC curve receiver operating characteristic curve, SMU Na/K second morning urine sodium/potassium ratio, K potassium, PAC plasma aldosterone concentration, AUC area under the ROC curve, CI confidence interval

Participants with high PACs had values ranging from 24.3 to 39.2 ng/dL. Figure 2 shows the ROC curves for the association of the SMU Na/K with a high PAC stratified by sex. The area under the ROC curve (AUC) for the relationship between the SMU Na/K and a high PAC was 0.77 (95% CI: 0.59–0.95) in men and 0.64 (95% CI: 0.36–0.93) in women. After age and serum K concentration were added, the AUC values became 0.80 (95% CI: 0.65–0.96) and 0.82 (95% CI: 0.69–0.95) in men and 0.64 (95% CI: 0.36–0.93) and 0.80 (95% CI: 0.61–1.00) in women, respectively. In men, a SMU Na/K <1.0 (mmol/mmol) predicted a high PAC, with a sensitivity of 45.5%, a specificity of 97.9%, a positive predictive value of 71.4%, and a negative predictive value of 94.1% (Table 3). The positive predictive values for a SMU Na/K <2.0 and <3.0 were much lower than that of a SMU Na/K <1.0.

**Table 3:** Sensitivities and specificities of SMU Na/K for high plasma aldosterone concentration (≥90th percentile) among men

	SMU Na/K <1.0 (95% confidence interval)	SMU Na/K <2.0 (95% confidence interval)	SMU Na/K <3.0 (95% confidence interval)
Sensitivity	45.5% (16.7–76.6)	63.6% (30.8–89.1)	90.9% (58.7–99.8)
Specificity	97.9% (92.7–99.7)	64.9% (54.6–74.4)	40.2% (30.4–50.7)
PPV	71.4% (29.0–96.3)	17.1% (7.2–32.1)	14.7% (7.3–25.4)
NPV	94.1% (87.5–97.8)	94.0% (85.4–98.3)	97.5% (86.8–99.9)

*PPV* positive predictive value, *NPV* negative predictive value, *SMU Na/K* second morning urine sodium/potassium ratio

The sensitivity analysis, from which participants with low sodium excretion (≤10th percentile value) were excluded, showed that the AUCs for the relationships between the SMU Na/K and a high PAC were 0.67 (95% CI: 0.46–0.87) in men and 0.62 (95% CI: 0.33–0.90) in women (Supplementary Figure).

## Discussion

We proposed a screening method using the spot urinary Na/K ratio to detect a high PAC among individuals with hypertension. In summary, the SMU Na/K had a significant inverse association with the PAC in hypertensive men but not in women. The AUC for the relationship between the SMU Na/K and a high PAC (≥90th percentile) was 0.77 (95% CI: 0.59–0.95) in men and 0.64 (95% CI: 0.36–0.93) in women. When the cutoff point was set to 1.0, the SMU Na/K could predict a high PAC, with a sensitivity of 45.5%, a specificity of 97.9%, a positive predictive value of 71.4%, and a negative predictive value of 94.1% in men. The positive predictive value was lower when the cutoff point was set at 2.0 and 3.0 than when it was set at 1.0. Thus, a low SMU Na/K could be a surrogate marker for a high PAC in men with hypertension. To the best of our knowledge, this is the first report to evaluate the association of the spot urinary Na/K ratio with a high PAC.

The consideration of hypokalemia as a symptom of hyperaldosteronism in the diagnosis of primary aldosteronism has been proposed. However, fewer than half of patients with primary aldosteronism have hypokalemia, according to a recent international multicenter study [17]. A suboptimal method for detecting primary aldosteronism suggested in previous studies is the use of the SUSPPUP index, which is calculated as the serum sodium to urinary sodium ratio divided by the (serum potassium) [2] to urinary potassium ratio [18, 19]. However, the reliability of the SUSPPUP index was unclear in a validation study [20]. Physiologically, the SUSPPUP is an index combining the urinary Na/K ratio and serum K level because the serum Na level does not usually change as a result of secondary hypertension. Both the urinary Na/K ratio and serum K level indicate a high PAC; thus, the SUSPPUP index can detect hyperaldosteronism but not primary aldosteronism or ARR. In the clinical setting, however, the independent assessment of the urinary Na/K ratio and serum K level may be more feasible than the calculation of the SUSPPUP index, which is complex.

The urinary Na/K ratio is easy to obtain, inexpensive, practical, and readily available in general outpatient clinics. It has been introduced as a biological marker of dietary quality because it indicates the balance between Na and K intake. Individuals who consume too much sodium and limited fruit and vegetables usually have a high urinary Na/K ratio. Dietary intervention focusing on reducing blood pressure could lower the urinary Na/K ratio in community-dwelling hypertensive men [21]. Previous reports have demonstrated associations of a higher Na/K ratio with higher risks of hypertension [22], CVD [23, 24], and mortality [24, 25]. In this context, a low urinary Na/K ratio should be a favorable sign. However, some people with sustained or progressive hypertension have a low urinary Na/K ratio even though they do not restrict their sodium intake or consume large quantities of fruit and vegetables. Our findings suggest that such individuals may have hyperaldosteronism if they are male. This insight may prevent clinicians from missing inappropriate aldosterone excretion among hypertensive men with a low Na/K ratio.

In our study, we defined a high PAC as ≥90th percentile of PAC. Although the PAC cutoff point for identifying primary aldosteronism in Japan has been discussed in previous reports [10, 26], we could not set specific cutoff points for the following two reasons. (1) In this study, we aimed to detect hyperaldosteronism not just primary aldosteronism. (2) Prioritizing feasibility, we measured the PAC in a seated position without a 30-min rest. We suggest that hypertensive men with extremely low SMU Na/K (e.g., <1.0 mmol/mmol) should undergo detailed examinations for hyperaldosteronism.

There was a significant interactive effect of the log SMU Na/K and sex on the PAC. Unlike in men, we found no significant association between the SMU Na/K and the PAC in women. Although the reason for this is unclear, we considered several possible explanations, which are as follows. First, the sample size of women was smaller than that of men. Thus, it might have been difficult to detect an association between the SMU Na/K and the PAC in women. Second, women consumed more vegetables and legumes than men, and these foods have high K levels (Table 1). It has been reported that a high frequency of the intake of fruit and vegetables is associated with a low urinary Na/K ratio [27]. Vegetable intake might interact with the association between the SMU Na/K and the PAC; thus, this association might not be detectable in women. Third, the homeostasis of Na and K may differ according to sex. The urinary Na level was significantly lower in men with a high PAC than in those without a high PAC; the same observation was not made in women (Supplementary Table 1). Morimoto reported that the rate of sweating among women was lower than that among men who were exposed to heat [28]. Women might sweat less and excrete less Na via the skin surface than men; thus, the urinary Na/K ratio might not become extremely low in women. Additionally, the serum K level in women with a high PAC was significantly lower than that in women without a high PAC (Supplementary Table 1). The AUC became significant when the serum K level was included in the model for women (Fig. 2). Although this could be a chance finding owing to the small sample size, a combination of the SMU Na/K and serum K level might improve the detection of hyperaldosteronism in women. Hypokalemia is a classic characteristic of hyperaldosteronism [29]; therefore, it is important to assess the serum K level together with the SMU Na/K. Further studies with large sample sizes are needed to test this hypothesis.

This study has several limitations. First, this was a small-sample study conducted at a single center. Thus, the feasible cutoff point for the SMU Na/K and the reasonable range for a high PAC for the detection of hyperaldosteronism should be validated in a future study. Second, our findings were obtained by a single measurement of the spot urinary Na/K ratio. Although the SMU Na/K might be similar to the 24-h urine Na/K ratio, the value in a single spot urine test includes random error compared with the 24-hour Na/K ratio in individuals. Repeated measurements of the spot urine Na/K ratio have been reported to provide a good estimate of the 24-h Na/K ratio [30, 31]. Considering the feasibility of implementation, repeated measurements of the SMU Na/K with a self-monitoring device could improve the detection of hyperaldosteronism [32]. Further study is needed to evaluate the usefulness of applying these findings and methods. Third, the association between a low urinary Na/K ratio and a high PAC may reflect compensation by the PAC in response to low sodium intake. Sodium restriction is usually recommended for hypertensive individuals to lower their blood pressure; however, biologically, this causes an increase in the PAC [33]. To validate how the present result was affected by “reverse causality,” a reliable way of measuring individual sodium intake is essential. Unfortunately, we were unable to collect 24-hour urine (the gold-standard method of estimating individual sodium intake) in this study. However, we recruited participants with persistent hypertension to exclude individuals with a high PAC resulting from sodium restriction. We also performed a sensitivity analysis, from which we excluded participants who might have had low sodium intake, as determined using the Kawasaki equation [14] (Supplementary Figure). The results obtained were similar to those of the main analysis, although the AUC of the ROC curve was slightly smaller for men. However, it was difficult to remove the influence of sodium intake on PAC in the present study, and therefore, we should be aware of the possibility of reverse causality when interpreting the association between the urinary Na/K ratio and the PAC.

## Conclusion

The urinary Na/K ratio in second morning urine was inversely associated with the PAC in hypertensive men but not in women. A low urinary Na/K ratio could be a surrogate marker for the detection of a high PAC in hypertensive men.

## Supplementary information

Supplementary Figures

Supplementary Table 1

Supplementary Table 2
