# Supplementary material for: Urinary sodium/potassium ratio as a screening tool for hyperaldosteronism in men with hypertension
Source: Hypertens Res. 2021 May 17;44(9):1129–37. doi: 10.1038/s41440-021-00663-9 (PMC8418986; doi:10.1038/s41440-021-00663-9)
Supplement: Supplementary file 1 — Supplementary Figures [file 41440_2021_663_MOESM1_ESM.pptx]

## Slide 1
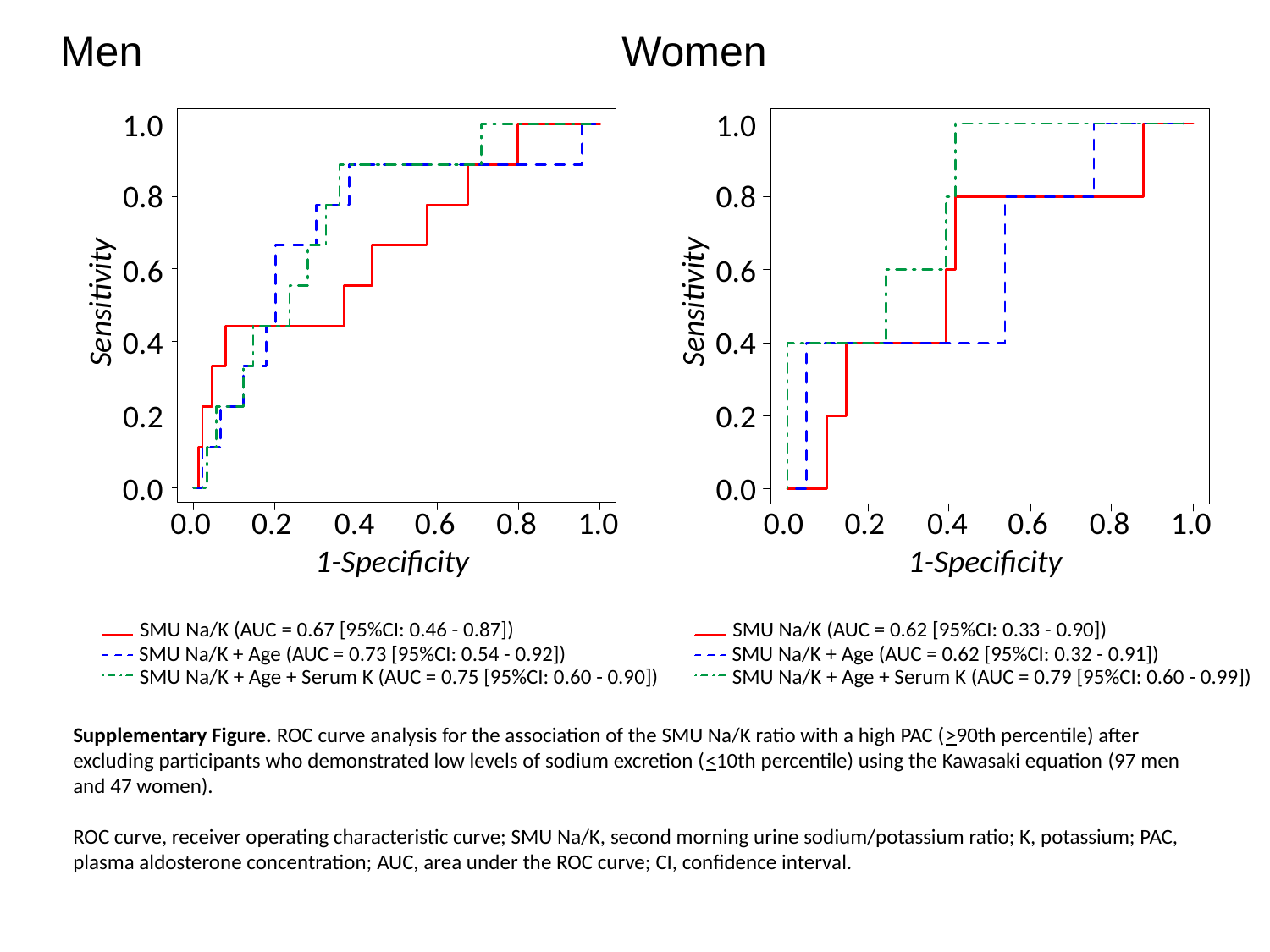

Men
1.0
0.8
0.6
Sensitivity
0.4
0.2
0.0
0.0
0.2
0.4
0.6
0.8
1.0
1-Specificity
SMU Na/K (AUC = 0.67 [95%CI: 0.46 - 0.87])
SMU Na/K + Age (AUC = 0.73 [95%CI: 0.54 - 0.92])
SMU Na/K + Age + Serum K (AUC = 0.75 [95%CI: 0.60 - 0.90])
Women
1.0
0.8
0.6
Sensitivity
0.4
0.2
0.0
0.0
0.2
0.4
0.6
0.8
1.0
1-Specificity
SMU Na/K (AUC = 0.62 [95%CI: 0.33 - 0.90])
SMU Na/K + Age (AUC = 0.62 [95%CI: 0.32 - 0.91])
SMU Na/K + Age + Serum K (AUC = 0.79 [95%CI: 0.60 - 0.99])
Supplementary Figure. ROC curve analysis for the association of the SMU Na/K ratio with a high PAC (>90th percentile) after excluding participants who demonstrated low levels of sodium excretion (<10th percentile) using the Kawasaki equation (97 men and 47 women).
ROC curve, receiver operating characteristic curve; SMU Na/K, second morning urine sodium/potassium ratio; K, potassium; PAC, plasma aldosterone concentration; AUC, area under the ROC curve; CI, confidence interval.
